# The role of immunotherapy in early-stage and metastatic NSCLC

**DOI:** 10.3389/pore.2024.1611713

**Published:** 2024-07-04

**Authors:** Attila Lieber, Attila Makai, Zsuzsanna Orosz, Tamás Kardos, Susil Joe Isaac, Ilona Tornyi, Nóra Bittner

**Affiliations:** ^1^ Department of Pulmonology, University of Debrecen, Debrecen, Hungary; ^2^ National Koranyi Institute of Pulmonology, Budapest, Hungary

**Keywords:** NSCLC, PD-(L)1, ICI, perioperative, metastatic

## Abstract

In the past decade we have seen new advances and thus remarkable progress in the therapeutic options for non-small cell lung cancer (NSCLC). Among cytostatic therapies with new approaches in molecularly targeted therapies, we see new developments in a wide range of applications for immunotherapies. In this review we discuss the new potential modalities for the use of immune checkpoint inhibitors (ICIs) in the frontlines, including in early-stage (perioperative) and metastatic settings. The perioperative use of ICIs in both neoadjuvant and adjuvant settings may show benefits for patients. In early-stage NSCLC (from stage IIB and above) a multimodality approach is recommended as the gold standard for the treatment. After surgical resection platinum-based adjuvant chemotherapy has been the standard of care for many years. Based on the benefit of disease-free survival, the approval of adjuvant atezolizumab and adjuvant pembrolizumab was a significant breakthrough. In the metastatic setting, the use of immune checkpoint inhibitors with chemotherapy, regardless of PD-L1 expression or ICI alone (PD-L1 expression equal to or greater than 50%) also improves overall survival and progression-free survival.

## Introduction

Non-small cell lung cancer (NSCLC) is the leading cause of cancer death worldwide, with more than 40% of patients diagnosed at stage IV [1]. The management approach for NSCLC primarily relies on the stage of the disease. However, advances in molecular pathology diagnostics, targeted therapy, and immunotherapy are expanding the range of treatment options and enhancing the prospects for improved survival rates. Immunotherapy has altered the treatment approach for several malignancies over the past 6–8 years, with NSCLC being among the most impacted. Tumor cells frequently reduce the expression of immune surveillance-related proteins, shielding them from the host’s protective immune response [2]. Numerous approaches have been developed to boost the body’s immune system in its fight against cancer cells by targeting pathways that suppress immune responses. In typical circumstances, activated T cells carry a receptor called the programmed cell death-1 (PD-1) protein. PD-1 helps regulate immune responses to prevent them from becoming overly aggressive. Its counterpart, PD-L1, is found in both immune and tumor cells. The interplay between the PD-1/PD-L1 pathways plays a critical role in allowing tumors to evade the immune system. However, when this interaction is blocked, it reactivates T cell-mediated antitumor immunity, offering a survival advantage in various advanced and treatment-resistant cancers [3]. In total, seven immune checkpoint inhibitors (ICIs) have been approved in the United States (US) and Europe (EU) for the initial treatment of advanced or metastatic NSCLC. These ICIs include pembrolizumab, atezolizumab, cemiplimab, nivolumab + ipilimumab, and durvalumab + tremelimumab. Available treatment options include ICI monotherapy, combined ICI therapy, and ICI in conjunction with chemotherapy [4–16].

## Perioperative treatment

Immunotherapy has become a very important part of the perioperative treatment of NSCLC. As shown in the following studies, the addition of ICI to perioperative chemotherapy treatment is very promising. Some studies tried the ICI + ICI combination, but because of its higher toxicity, this study was canceled [17]. Immunotherapy has become an increasingly important component of the perioperative treatment of NSCLC. The *National Comprehensive Cancer Network* (NCCN) has made its recommendations regarding the treatment with atezolizumab or pembrolizumab in the adjuvant setting, and nivolumab + chemotherapy or pembrolizumab + cisplatin doublet therapy and postoperative therapy in the neoadjuvant-adjuvant setting for specific patient populations with NSCLC [18].

### Biomarkers

PD-L1 overexpression means a worse prognosis for the patients, namely, decreased disease-free survival (DFS) and overall survival (OS), which is clearly true in the cases of resected NSCLC tumors, so higher levels of PD-L1 indicate worse survival data. In the case of tumor mutation burden (TMB), the results are controversial as to whether they have a prognostic value for resected stage I-II NSCLC. Perioperative ctDNA analysis appears to be very useful in predicting event-free survival (EFS). Low preoperative and undetectable postoperative ctDNA levels mean better EFS [19]. From the perspective of response to therapy, in the IMpower010 trial in patients whose NSCLC tumor expressed PD-L1 more than 1%, better tumor regression was seen, but after deeper statistical analysis, it seems the high PD-L1 expressing (≥50%) group benefits the most in DFS. In contrast to the Keynote-091 study this benefit could not be demonstrated. A relatively new approach in the determination of minimal residual disease (MRD) is to use ctDNA analysis as another biomarker of perioperative ICI. After publications such as the results of the Mermaid studies we will see the place of this approach. Other potential future biomarkers such as blood TMB level, the ratio of lymphocytes that are infiltrating the tumor, and, e.g., KEAP1, STK11, and TP53 gene mutations may predict the benefit of ICI therapy [20].

### Neoadjuvant treatment

Some trials have examined the efficacy of neoadjuvant cisplatin-based chemotherapy, showing an increase in overall survival (HR 0.84) [21]. Objectivizing the efficacy of neoadjuvant therapy is easier compared to adjuvant treatment because the pathologic response can be seen directly in the surgical specimen. A complete pathologic response (pCR) which means that no living tumor cells can be seen in the surgical specimen was found to be more beneficial for survival [22]. Major pathologic response (MPR) is an important parameter, which is defined as 10% or less of living tumor cells compared to necrosis and stromal cells in resected tissue [23]. This definition of MPR has been set to predict OS in prospective treatment [24]. In cases where neoadjuvant therapy was chosen, the proposed risk of delay or cancellation of surgery due to treatment-induced adverse events (AE) or disease progression was also considered. A few trials of neoadjuvant immunotherapy treatment are presented that may provide clarity in some of these situations (Table 1).

**TABLE 1:** Clinical trials of perioperative immunotherapy for NSCLC.

Trial	ICI agent	Stage	pCR (ICI arm)	MPR (ICI arm) (%)	G3 or higher TRAE (ICI arm) (%)	Rate of surgery (ICI arm)
Checkmate 816	nivolumab	IB-IIIA (TNM 7)	24%	36.9	33.5	83.2%
NADIM	nivolumab	IIIA	63.4%	82.9	13.5	90.2%
NADIM II	nivolumab	IIIA and IIIB	37%	54	19	93%
Checkmate77T	nivolumab	II-IIIB	25.3%	35.4	78	32%
Impower030	atezolizumab	II, IIIA, or select IIIB (T3N2)	only the study design was available			
Neotorch	toripalimab	II-III	24.8%	48.5	64.3	not known
Keynote-671	pembrolizumab	II, IIIA or IIIB (N2)	18.1%	30.2	44.9	81.2%
AEGEAN	durvalumab	IIA-IIIB (N2)	only the study design was available			

TRAE: treatment-related adverse event, ICI: immune checkpoint inhibitor, pCR: pathologic complete response, MPR: major pathologic response.

In the **Checkmate 816** trial the examined patients had stage IB-IIIA NSCLC (TNM 7th edition), no previous anticancer therapy, PD-L1 expression determined, ECOG 0-1, while EGFR and ALK alterations were excluded. In the experimental arm they received nivolumab + chemoterapy (3 cycles every 3 weeks) or chemotherapy alone in the control arm, equally distributed. In total, 83.2% of the experimental arm and 77.8% of the control group had R0 resection. Surgery was canceled only in 1.1% and 0.6% of patients due to adverse events. The follow-up period was not less than 21 months in this study. The median EFS was 31.6 months (statistically not reached) in the experimental arm and 20.8 months in the control arm. HR for recurrence, death or progression was 0.63. The greatest benefit in EFS was observed in stage IIIA, with PD-L1 expression of more than 1%, a non-squamous histological type and a carboplatin component in the treatment. PCR was 24% in the experimental arm and 2.2% in the control arm. The MPR was 36.9% in the experimental arm and 8.9% in the control arm. The statistically calculated median OS was not reached in either group (HR 0.57). After exploratory analysis, it became obvious that in patients with a complete pathologic response, the median EFS was significantly better in the experimental arm (26.6 vs. 18.4 months). The ctDNA clearance was higher in the experimental arm (56%) than in the control arm (35%), which correlated with the differences in EFS between the two groups. The investigators also found a positive correlation between pCR and ctDNA clearance. Grade 3 or higher side effects were almost equal in the two groups (33.5% and 36.9%) [25, 26].

### Neoadjuvant + adjuvant treatment

**NADIM** is a single-arm study that is now in phase II. Its aim was to measure the role of the ctDNA level in prognosis. The researchers enrolled stage IIIA NSCLC patients who were likely to have the potential for surgical removal of the tumor. The treatment was carboplatin doublet with paclitaxel plus nivolumab in the neoadjuvant setting, followed after surgery by nivolumab for 1 year (at a known dose). In total, 90.2% of the planned population underwent surgery. OS was 81.9% in the overall treated group and 91% in the nivolumab group at 36 months follow-up. A total of 63.4% of patients had pCR, including 82.9% of patients with MPR. The researchers found that neither TMB nor PD-L1 were independent predictors of long-term survival. Before treatment, low ctDNA levels were associated with longer progression-free survival (PFS) and OS (HR: 0.20) and zero ctDNA levels after the adjuvant treatment were associated with improved PFS and OS (HR: 0.26). Treatment-related adverse events (TRAEs) of grade 3 or higher were observed in 13.5% of patients [27].

**NADIM II** is a phase II trial that enrolled stage IIIA and IIIB NSCLC patients. They were randomly assigned in a 2:1 ratio to receive 3 cycles of nivolumab with paclitaxel + carboplatin (experimental arm), and after surgery (R0 resections) mono nivolumab 4 weekly for 6 months. The chemotherapy alone (3 cycles of paclitaxel + carboplatin) control group, before and after surgery received paclitaxel and carboplatin. PCR was 37% in the ICI arm and 7% without ICI treatment (this benefit was observed more in patients whose tumor expressed more than one percent PD-L1). The MPR was 57% vs. 14%. At 24 months PFS was 67.2% in the ICI arm and 40.9% in the only chemotherapy arm (HR 0.47). At the same follow-up time, OS was 85.0% in the ICI arm and 63.6% in the non-ICI arm (HR 0.43). In total, 93% of patients in the ICI arm and 69% of patients in the non-ICI group underwent surgery. One surgery was canceled because of ICI-related adverse events. Grade 3 or higher grade side effects were noted in 19% of patients. CtDNA analysis was also regularly performed in 66% of patients before and after neoadjuvant treatment. Pretreatment ctDNA levels were correlated with tumor size. After neoadjuvant treatment, ctDNA was negative in 67% of patients in the ICI arm and in 44% of patients in the non-ICI arm [28].

**Checkmate 77T** is a phase III trial that is currently in its interim analysis phase. The investigators are evaluating nivolumab with platinum-doublet chemotherapy (4 cycles) followed by surgery and nivolumab (1 year), or placebo with platinum-doublet chemotherapy (4 cycles) followed by surgery and placebo (1 year) in R0 resected stage II-IIIB, NSCLC, ECOG 0-1, EGFR/ALK wild-type, PD-L1 any expression patients. In this study, the follow-up time was no less than 15.7 months. At this time point, the median EFS in the ICI + chemotherapy + adjuvant group was observed. In the ICI arm, the median EFS is 28.9 months, compared to 18.4 months in the chemotherapy + placebo arm (HR: 0.58). PCR rates were improved as well (25.3% vs 4.7%), and MPR rates were higher in the ICI group (35.4% vs 12.1%). In total, 78% and 77% of patients in the two groups underwent definitive surgery and R0 resection was achieved in nearly 90% of cases. Grade 3 or higher adverse events were 32% and 25%, respectively [29].

**Impower030** is a phase III trial in which the study population was potentially resectable stage II, IIIA, or select IIIB (T3N2) NSCLC patients with ECOG 0-1 performance status, EGFR wild-type, and without ALK translocation, but PD-L1 expression was not measured. The subjects received neoadjuvant atezolizumab or placebo plus chemotherapy (platinum-doublet). After surgery patients in the experimental arm received atezolizumab treatment for 16 cycles or until recurrence or unacceptable toxicity, and patients in the control arm received the best supportive care and follow-up. The results of this study are not yet available [30].

**Neotorch** (phase III) enrolled patients with stage II/III, NSCLC, without EGFR or ALK alterations. Patients received 3 cycles of toripalimab or placebo with chemotherapy, and 13 cycles of toripalimab or placebo treatment after resection Q3W. While the trial is ongoing and EFS has not yet been reached in the ICI arm, it is 15.1 months in the control group (HR 0.40); the outcome is quite promising after 18 months of follow-up. The PCR was higher in the ICI group (24.8% vs. 1%), and MPR was also better in the toripalimab group (48.5%) versus 8.4% in the control group. AEs (grade 3 or higher) were almost equal, with 63.4% in the ICI group and 54.0% in the control group [31].

**Keynote-671** is a phase III study in which only interim analysis is available at the moment. The trial enrolled patients eligible for R0 resection of stage II, IIIA or IIIB (N2) NSCLC. Patients received a total of 4 cycles of neoadjuvant pembrolizumab or placebo + cisplatin doublet therapy every 3 weeks and pembrolizumab or placebo (13 cycles) after surgery. With a median follow-up of 25.2 months EFS at 24 months was 62.4% in the ICI arm and 40.6% in the placebo group (HR 0.58). The calculated 24-month OS was 80.9% in the ICI arm and 77.6% in the control arm (not statistically significant). The PCR was 18.1% in the ICI arm and 4.0% in the control arm. MPR was 30.2% in the ICI arm and 11.0% in the control arm. In total, 81.2% of participants in the ICI arm and 79.4% of participants in the control arm underwent surgery. Grade 3 or higher toxicity was 44.9% in the ICI group and 37.3% in the control group. Toxicity that led to cancellation occurred in 12.6% of the patients in the ICI arm and 5.3% of the patients in the control group. Subgroup analysis showed that patients who are smokers, stage III have more benefit in EFS and in contrast to other trials nonsquamous phenotypes have benefitted in terms of EFS compared to squamous phenotypes. Every PD-L1 expression subgroup has benefitted in terms of EFS, but the biggest benefits were observed in the high (TPS>50%) expression group (HR: 0.42) [32].

**AEGEAN** will be a phase III trial, but only the study design has been published. Eligible patients are: no prior oncotherapy, candidates for complete resection, stage IIA to select (N2) IIIB NSCLC (according to TNM 8), without EGFR mutations or ALK rearrangement, with measured PD-L1 expression. Eligible participants will receive durvalumab or placebo on platinum doublet treatment and durvalumab or placebo after resection for 12 cycles. The primary endpoints are pCR and EFS [33].

**The combination of IO + IO** in the neoadjuvant setting is an exciting area of inquiry. It has greater immune activation and increased T-cell infiltration into the tumor tissue with ipilimumab + nivolumab therapy but surgical outcomes are not better. A study evaluating the effect of the neoadjuvant ipilimumab and nivolumab combination was stopped early (only 9 participants were selected) because of high rates of toxicity and progression, which canceled resection [17]. Despite a better response, the addition of ipilimumab seems to have a greater risk of serious adverse events leading to the cancellation of a potential complete resection of the tumor [34].

In the neoadjuvant setting there is a two-step approach, the only preoperative treatment as Checkmate816 and after neoadjuvant treatment and surgical resection with “adjuvant” immunotherapy treatment in various cycles. Assessing the efficacy of this treatment is straightforward since the pathological response can be objectively observed in surgical samples. The PCR rate is unexpectedly high after neoadjuvant chemo + ICI therapies (e.g., 18%, 24%, and 37%), and MPR (including pCR as well) is also very promising (37%, 57%, and even 82% were observed) compared to the chemotherapy-only group, where rates are around 2.2% and 4%. It is too early to draw conclusions from the survival data because of the short follow-up time, but the results that have already been presented are very promising. Another question is: were many surgeries canceled because of the high toxicity of this combination of neoadjuvant treatments? The answer is no. The Checkmate816 trial served as the prototype for the neoadjuvant ICI + chemo combination, in this trial, 1%–2% of patients were not operated on because of adverse events, and in NADIM II there are no patients in the same conditions. While in the majority of the trials stage IB-IIIA patients were enrolled, the greatest DFS survival benefit from ICI therapy was seen in stage IIIA patients with more than 1% PD-L1 expression, nonsquamous histology and those who received carboplatin treatment. Surprisingly, this same finding was not found in Keynote-671 in the nonsquamous histology type. The follow of the ctDNA level happened in the trials and they found positive correlation between complete ctDNA clearance and pCR. This approach is very promising and new in both the adjuvant and neoadjuvant settings as well.

## Adjuvant therapy

### Why is adjuvant immunotherapy useful?

Many trials have compared resection alone with surgery and adjuvant cisplatin-based doublet therapy. In a meta-analysis of adjuvant cisplatin-based doublet therapy, the addition of chemotherapy improved OS (HR 0.89) with a 5.4% risk of disease recurrence [35].

Despite the limited benefit of adjuvant therapy in overall survival (OS), it is administered when indicated. In such cases, the goal of adjuvant therapy is to eradicate potential micrometastases and prevent recurrence of lung cancer [20]. Given the very good results from ICI therapy in metastatic NSCLC, the question arose as to whether we could achieve similarly good results with ICI therapy in the adjuvant setting. First, the background of the potential effectiveness and the possible targets of adjuvant ICI therapy were examined. It is known that cancer-related immune dysfunction can occur after surgical resection and may be a theoretical target of ICI, since the immune system reacts to surgery with various inflammatory responses and metabolic events [36, 37]. The surgical procedure itself, which includes trauma, blood loss, and hypothermia, may result in immunosuppression. More specifically, Th2 immunity increases in the postoperative period causing the release of growth factors and stress hormones [38]. These changes also lead to the expansion of myeloid-derived suppressor cells, M2 macrophages and T regulatory cells [36], which in turn will lead to suppression of the cellular immune system, resulting in higher expression of PD-L1 and CTLA-4. The altered PD-L1 and CTLA-4 expression appear to make adjuvant ICI treatment highly beneficial and effective in this setting [39]. The synergistic effect of combined ICI and chemotherapy is more effective in the destruction of MRD after surgical resection [40].

#### Clinical trials

Various immune checkpoint inhibitors have been and are still under examination to prove their effectiveness in the adjuvant setting. Some of these trials are presented below.

**BR31/IFCT1401** is a phase III, double-blind trial. The investigators enrolled patients with completely removed stage IB-IIIA NSCLC (according to TNM 7th edition). This trial started in 2014 and is planned to finish in 2024 [41]. It is planned to enroll 1,415 patients and EGFR or ALK alterations are not part of the exclusion criteria. After R0 resection and completion of adjuvant chemotherapy, patients received durvalumab or placebo for 1 year. The primary endpoint was DFS for NSCLC participants with PD-L1 expression (greater than 25%) and without EGFR mutations or ALK rearrangements [20].

In **Impower010** 1,280 patients were enrolled in this phase III trial. Eligible patients for the study included those who had undergone R0 resection, and were in stage IB-IIIA according to the TNM 7 stage and ECOG 0-1. Participants were administered either 16 cycles of adjuvant atezolizumab or received best supportive care following cisplatin doublet chemotherapy (consisting of pemetrexed, docetaxel, vinorelbine, or gemcitabine) for 1-4 cycles. The median follow-up period was 32.8 months. Data processing focused on stage II-IIIA patients, where the primary endpoint was based on PD-L1 expression. Specifically, it looked at the difference in DFS between patients with PD-L1 expression greater than 1%. The analysis revealed a stratified HR for DFS of 0.66 when comparing the ICI group to the control group. In the overall study population (regardless of PD-L1 expression), the difference between the intent-to-treat group and the control group was observed with an HR of 0.79 for DFS. In the stage II–IIIA population with PD-L1 expression ≥1%, the 3-year DFS rates were 60% in the ICI group and 48% in the control group. In the overall population of stage II–IIIA participants, the 3-year DFS rates were 56% in the ICI group and 49% in the control group. The 5-year DFS rates could not be measured because this was an interim analysis. For the secondary endpoint of DFS in patients whose tumors had high PD-L1 expression (>50%), the unstratified HR was 0.43. In further exploratory analyses in stage II–IIIA participants whose tumors expressed 1%–49%, PD-L1 the unstratified HR was 0.87, and in patients with PD-L1<1%, the unstratified HR was 0.97. Unfortunately, OS data were immature in this analysis. Grade 3 or higher grade toxicity occurred in 22% of participants who received ICI and 12% in the control group. Looking at the risk of disease recurrence, new primary tumor appearance or death, it was reduced by 34% with ICI compared to the best supportive care in the PD-L1>1% expressing group and by 21% in the overall patient population. The DFS benefit with atezolizumab was of course highest in patients with tumors expressing PD-L1 >50%, but surprisingly a high DFS benefit could not be seen in the 1%–49% PD-L1 expression subgroup. As EGFR or ALK alterations were not exclusion criteria, it is an interesting question whether there is a difference in the DFS data in these patients. The data indicate that patients with driver mutations did not show a difference in DFS compared to patients without driver mutations. However, these findings should be interpreted with caution due to the low number of participants [42].

**PEARLS/Keynote-091** is a triple-blind trial and has now reached its interim analysis. In total, 1,177 participants were enrolled with R0 resected NSCLC, stage IB-IIIA (according to TNM 7), ECOG 0-1 performance status, any verified PD-L1 expression level, and known EGFR and ALK alterations was not a requirement for inclusion. Patients who received adjuvant chemotherapy received ICI treatment within 3–12 weeks after the last dose of chemotherapy. The trial did not exclude patients who did not receive adjuvant chemotherapy previously; therefore, their ICI treatment began within 12 weeks after surgery. Eligible participants received pembrolizumab or placebo every 3 weeks until disease recurrence or intolerable adverse events (up to a maximum of 18 cycles). Crossover was not possible in this trial. Median DFS was 53.6 months in the ICI arm and 42.0 months in the control arm (HR 0.76). When examining the high PD-L1 expressing population, the median DFS was not statistically met in either the ICI arm or the control arm (HR 0.82). It was surprising that the benefit of DFS for ICI was not detected in the PD-L1 > 50% group which is likely due to the relative benefit of ICI treatment increasing with increasing PD-L1 expression in the setting of locally advanced or metastatic NSCLC. Despite this, median DFS in the ICI arm was numerically higher in the PD-L1 >50% population compared with the lower (1%–49% or <1%) PD-L1 expressed populations. What we did not expect was that median DFS in the control arm was also numerically improved in the PD-L1> 50% population compared with the lower (1%–49% and <1%) expressing group. These statistical imbalances probably occurred due to the short follow-up period. As this was an interim analysis, the median OS data are immature (HR 0.87). Grade 3 or higher side effects occurred in 34% of patients in the ICI arm and 26% of patients in the placebo group [43].

**ANVIL** is an ongoing trial that started in May 2016 and is planned to be completed in July 2024. Unfortunately, only the study design is accessible. In total, 903 patients were enrolled with operated NSCLC (stage IB-IIIA, according to TNM7). Tumors with an EGFR mutation or ALK rearrangement were excluded. Adjuvant chemotherapy and radiotherapy were not mandatory. Randomized patients received adjuvant nivolumab or were observed for 1 year. The primary endpoints are DFS and OS. The secondary endpoint is the incidence of AEs and their severity [44].

**Alchemist** is a National Cancer Institute clinical trial platform for biomarker analysis of high-risk resected NSCLC that supports different randomized trials of new adjuvant therapies within the National Clinical Trials Network (NCTN). It includes a screening trial that enrolled participants with stage IB-IIIA (according to TNM7) who underwent R0 surgical resection, and had tissue and blood samples collected for analysis of EGFR and ALK alterations and PD-L1 expression. After the results patients were enrolled to receive adjuvant erlotinib, adjuvant crizotinib or adjuvant nivolumab after adjuvant chemotherapy [45].

**ACCIO** is a new three-arm trial in the Alchemist portfolio that started in June 2020. The study design is very interesting as it contains 3 arms (Arm A: 4 cycles of platinum doublet and observation, Arm B: 4 cycles of platinum doublet treatment + sequential pembrolizumab therapy for 16 cycles and Arm C: 4 cycles of platinum doublet chemotherapy + pembrolizumab with maintenance pembrolizumab of maximum 12 cycles) with or without postoperative radiotherapy (when needed). Stratification factors were NSCLC histologic type, any stratified PD-L1 expression, smoking habits and stage IB and II vs. IIIA. The primary endpoints are DFS and OS and this study design allowed for the secondary objective of comparing the primary DFS and OS endpoints between arms B and C in the overall population [46].

**MERMAID-1** is an interesting phase III parallel-arm, placebo-controlled, double-blind, multicenter trial that was initiated in July 2020. Its estimated completion date is September 2026. Patient enrollment criteria are no EGFR or ALK alterations, stage II-III (according to TNM 8), R0 resection, ECOG 0-1, NSCLC histology, and stratified PD-L1 status. Minimal residual disease (MRD) status was determined by ctDNA analysis of blood samples collected 3–4 weeks after resection. Patients will be randomized 1:1 to get durvalumab or placebo, plus chemotherapy, for 12 weeks. Treatment continues with durvalumab or placebo, until week 48 or disease recurrence. It is very exciting that the primary endpoint (DFS), is determined by the measurement of MRD. Secondary endpoints will be: DFS, DFS in the minimal residual disease positive analysis set; and FAS (blinded independent central review) and OS in the MRD+ analysis set and FAS [47].

**MERMAID-2 was** launched in November 2020 and is planned to finish in October 2027. Enrolled patients were selected according to the MERMAID-1 requirements (R0 resected, stage II-III (TNM 8), no EGFR or ALK alterations). MRD is monitored by ctDNA levels from plasma samples and patients without visible recurrence but MRD+ with ctDNA levels are selected in this trial, so subjects with definitive therapy (R0 resection + optional neoadjuvant and/or adjuvant therapy) are elected in a 96- week follow-up phase, which means that patients will be examined regularly for MRD with ctDNA level measurement of blood samples. MRD-positive participants are evaluated with negative imaging (no visible tumor) and measured PD-L1 expression to determine eligibility for the trial. Eligible subjects receive durvalumab or placebo for up to 2 years or until disease recurrence. The primary endpoint is DFS in participants whose tumor expresses PD–L1 ≥1%. Secondary endpoints are DFS in the full analysis set, PFS, OS, quality of life questionnaires and rate of side effects [48].

**Adjuvant-designed NADIM** is an open-label trial that started in January 2021. Enrolled patients receive four cycles of chemotherapy + nivolumab and after 6 cycles of nivolumab or chemotherapy alone 4 cycles. The primary endpoint is DFS. Similar studies have been launched with toripalimab and canakinumab (interleukin-1β blocker) [20].

In the adjuvant setting, many trials are still ongoing; some have only reached the study design and interim analysis phase. The enrollment criteria are similar in many ways, but there are also some differences. In all trials patients were enrolled with completely resected NSCLC with stage II-IIIA (TNM 7 or 8). Impower010 and Keynote-091 did not exclude EGFR and ALK alterations. In Impower010 adjuvant cisplatin base doublet chemotherapy was mandatory while in Keynote-091 it was not, but previous radiotherapy or chemotherapy was prohibited [42, 43]. Despite the relatively immature data, each trial showed a significant EFS benefit compared to adjuvant chemotherapy. In Impower010 the greatest DFS benefit could be seen in the PD-L1 expression ≥50% group, but interestingly in Keynote-091 the PD-L1 ≥50% group did not show this benefit despite the results with pembrolizumab in metastatic disease [42, 43]. There were two interesting study designs that are very promising and may become the basis for future treatments. In ACCIO, the three-arm study design allows a head-to-head comparison of adjuvant chemotherapy versus adjuvant chemotherapy combined with ICI in both sequential and synchronous settings [46]. In Mermaid I and II the use of ctDNA measurement to determine the MRD before adjuvant treatment is quite exciting, but the results of the study are still ongoing [47, 48].

## Advanced and metastatic stages

### First line

In stage IV lung cancer, the advanced stage itself may be diagnosed primarily at metastatic sites. In practice, we are generally dealing with small samples. All patients diagnosed with stage IV NSCLC (nonsquamous, squamous) should be tested for driver mutations and for PD-L1 expression. When deciding on the treatment plan for a patient without an oncogene driver, several factors need to be considered. These factors include the histology, the tumor genotype, the level of PD-L1 expression, patient performance status (PS), any existing medical conditions (comorbidities), and the patient’s own preferences [49]. ICIs that focus on either PD-1 or PD-L1 have been incorporated into the standard clinical strategy for the treatment of NSCLC. Key phase III studies (Table 2) evaluating various anti-PD-(L)-1 drugs, either alone or in combination with chemotherapy, have established ICI as the primary first-line therapy for metastatic NSCLC without targetable genetic mutations. Despite the progress made, there are unresolved challenges that include determining the best treatment regimen for individual patients. To date, there has been no direct comparison of different ICI-containing therapies in the first-line setting [49, 50].

**TABLE 2:** Clinical trials of first line immunotherapy in advanced and metastatic NSCLC.

Trial	ITT treatment regimen vs. cht	N (patients)	mOS (months)	mPFS (months)
Keynote 024	pembrolizumab	305	30 vs. 14.2 HR 0.63	10.3 vs. 6.0 HR: 0.50
EMPOWER-Lung 1	cemiplimab	712	26.1 vs. 13.3 HR 0.57	8.1 vs. 5.3 HR 0.51
IMpower 110	atezolizumab	572	20.2 vs. 13.1 HR 0,59	8.1 vs. 5.0 HR 0.63
Keynote-189 (nonsquamous)	pembrolizumab/pemetrexed/platinum	616	22.0 vs. 10.6 HR 0.49 (at 5 years)	8.8 vs. 4.9 HR 0.52
Keynote-407 (squamous)	pembroliumab/(nab)-paclitaxel/carboplatin	559	15.9 vs. 11.3 HR 0.64	6.4 vs. 4.8 HR0.56
EMPOWER-Lung-3 (squam + nonsqu.)	cemiplimab + cht	466	21.9 vs. 13.0 HR 0.71	8.2/5.5 HR 0.56
IMpower130	atezolizumab + nab-paclitaxel + carboplatin	723	18.6 vs. 13.9 HR 0.79	7.0/5.5 HR 0.64
IMpower150 (nonsquamous)	atezolizumab + carboplatin + paclitaxel + bevacizumab	1,202	19.2 vs. 14.7 HR 0.78	8.4 vs. 6.8 HR 0.57
CheckMate227	nivolumab + ipilimumab	1739	17.1 vs. 13.9 HR 0.79	7.2 vs. 5.5 HR 0.58
CheMate 9LA	nivolumab + ipilimumab + cht (2 cycles)	719	15.6 vs. 10.9 HR 0.66	6.8 vs. 5.0 HR 0.70

### Checkpoint inhibitor monotherapy

The standard of care for patients with squamous- and nonsquamous NSCLC, who also have a high PD-L1 expression, now involves the use of single-agent ICI. This is now the first-line therapy for patients with PD-L1 ≥50% with no contraindications for ICI. The significance of tumor mutational burden (in both blood and tissue samples) as a predictive indicator of response to cancer immunotherapy in metastatic NSCLC patients is still unclear. While the predictive utility of tumor mutational burden appears to be somewhat limited when it comes to patients receiving a combination of cancer immunotherapy and chemotherapy, recent data indicate that it may have a more meaningful predictive role in the context of immunotherapy alone, without the addition of chemotherapy [51]. If the choice is to use a single checkpoint inhibitor as a standalone treatment, either pembrolizumab, atezolizumab, or cemiplimab can be considered suitable options. The first evidence of enhanced survival outcomes emerged from investigations of NSCLC patients who had already experienced disease progression following platinum-based chemotherapy. This benefit was later extended to the frontline treatment of metastatic disease, whether used as a standalone therapy or in conjunction with chemotherapy. Additionally, this was also observed to be beneficial for patients with locally advanced unresectable disease [52]. A randomized trial has not directly compared the combination of a checkpoint inhibitor and chemotherapy with the use of a checkpoint inhibitor alone in individuals with high PD-L1 levels in NSCLC. Pembrolizumab, cemiplimab and nivolumab are anti-PD1 monoclonal antibodies, while atezolizumab and durvalumab are anti-PD-L1 monoclonal antibodies [18, 53]. To utilize pembrolizumab or cemiplimab as monotherapies in the initial treatment stage, it is necessary to have PD-L1 expression of more than 50%, which means that at least 50% of a minimum of 100 tumor cells (TCs) should show membrane expression of PD-L1. On the other hand, for nivolumab plus ipilimumab in the first line (although not approved by the European Medicines Agency), or pembrolizumab in the second line, a minimum of 1% PD-L1 expression on TCs is required [49].

### Pembrolizumab

The KEYNOTE-024 trial demonstrated that pembrolizumab, compared to platinum-based chemotherapy, significantly extended median PFS in previously untreated NSCLC patients with a PD-L1 tumor proportion score of at least 50% and no EGFR or ALK genetic alterations (10.3 vs. 6.0 HR 0.50). Importantly, the pembrolizumab group displayed an undefined median duration of response, suggesting the potential for long-lasting benefit. Additionally, the incidence of grade 3–5 TRAEs was less frequent in the pembrolizumab-treated patients. This study confirms the efficacy of pembrolizumab in the treatment of advanced NSCLC with high PD-L1 expression, highlighting its potential to provide better outcomes and improved tolerability compared to conventional chemotherapy [4]. Based on the 5-year median OS for pembrolizumab compared to chemotherapy (26.3 months vs. 13.4 months) and its 5-year OS rate (31.9% vs. 16.3%) the US Food and Drug Administration (FDA) approved the utilization of pembrolizumab as an initial treatment option in patients with advanced NSCLC [54, 55]. The KEYNOTE-598 study concludes that incorporating ipilimumab with pembrolizumab does not enhance efficacy and is linked to higher levels of adverse effects compared to using pembrolizumab alone in this group of patients (Grade 3 or higher TRAEs: 62.4% in pembrolizumab-ipilimumab recipients versus 50.2% in pembrolizumab-placebo recipients). Therefore, the findings do not support the use of the pembrolizumab-ipilimumab combination over the use of pembrolizumab alone in this context [56].

### Cemiplimab

Cemiplimab demonstrated enhanced OS and PFS in comparison to chemotherapy. The EMPOWER-Lung 1 trial compared the use of cemiplimab alone to the choice of chemotherapy made by the investigators in patients who were newly diagnosed with advanced NSCLC and tumor PD-L1 expression of at least 50%, along with no EGFR mutations or ALK or ROS1 fusions. This study involved 712 participants, 85% of whom were men. The cemiplimab group showed significantly longer median OS (26.1 months vs. 13.3 months) and PFS (8.1 months vs. 5.3 months) compared to the chemotherapy group (HR 0.57 and HR 0.51, respectively). Regarding adverse events, grade 3 or higher TRAEs were less prevalent in the cemiplimab group (18%) in comparison to the chemotherapy group (40%). In conclusion, this study supports the use of cemiplimab as a first-line monotherapy in patients with advanced NSCLC who have a high level of PD-L1 expression. Interestingly, combining chemotherapy with cemiplimab at disease progression showed significant clinical benefit, suggesting a potential novel treatment approach for these patients [11, 57].

### Atezolizumab

When considering atezolizumab as a first-line monotherapy treatment, criteria include PD-L1 expression of at least 50% on TCs or at least 10% on tumor-infiltrating immune cells [49]. The FDA approval of atezolizumab was based primarily on the outcomes of the IMpower 110 study. This research aimed to assess the efficacy and safety of atezolizumab compared to platinum-based chemotherapy. It was conducted as an initial treatment for patients with mNSCLC who exhibit PD-L1 expression. During the study 572 treatment-naïve patients were enrolled with metastatic nonsquamous or squamous NSCLC. In the high PD-L1 expression subgroup (205 patients), atezolizumab demonstrated a 7.1-month longer median overall survival (20.2 months vs. 13.1 months; HR for death 0.59) and an 8.1-month longer median PFS compared to chemotherapy (HR 0.63). Notably, grade 3 or higher TRAEs occurred in approximately 30% of atezolizumab patients and 53% of chemotherapy patients. These findings suggest that atezolizumab may be a more effective treatment option compared to traditional chemotherapy for these patients with NSCLC, regardless of histologic type [8]. The outcomes in terms of overall survival based on the degree of PD-L1 expression were consistent with those observed in the KEYNOTE-042 study comparing pembrolizumab to chemotherapy [5]. The main goal of the research was to evaluate the impact of OS on various population subgroups categorized by their PD-L1 expression levels. The study found varying degrees of OS benefit in these subgroups: those with PD-L1 >50%, PD-L1 >20%, and PD-L1 >1%. Notably, no significant PFS improvement was observed in patients with PD-L1 expression between 1% and 49% (HR: 0.92), leading to the approval of pembrolizumab monotherapy for subjects with PD-L1 expression above 50%. In both trials, individuals with high PD-L1 expression experienced the greatest benefit in terms of survival [5].

### Nivolumab, durvalumab

Unfortunately, in addition to the remarkable results described above, we also find studies that did not show promising results. The CheckMate026 study conducted a comparison between nivolumab and platinum-based chemotherapy in patients diagnosed with stage IV NSCLC who had PD-L1 expression levels greater than 5% in their tumor cells and did not have EGFR- or ALK-activating mutations. The results in this instance were unfavorable. Nivolumab had a shorter median PFS (4.2 months vs. 5.9 months for CT, HR: 1.15), similar OS (14.4 months vs. 13.2 months for CT, HR: 1.02), but significantly fewer severe adverse effects (17.6% vs. 50.6% for CT) [58]. Another negative result was seen in the MYSTIC trial which sought to evaluate the efficacy of durvalumab (anti- PD-L1), either alone or in combination with tremelimumab, compared to chemotherapy as the initial treatment in treatment-naive metastatic patients. The MYSTIC study did not meet its primary objective of showing a significant improvement in OS with durvalumab over chemotherapy, although durvalumab patients had a median OS of 16.3 months compared to 12.9 months for chemotherapy. Additionally, there was no statistically significant difference observed in PFS, which was a secondary endpoint of the study [59].

### Checkpoint inhibitors + chemotherapy

In accordance with international treatment recommendations for lung cancer, the standard approach for patients with PD-L1 expression levels below 50% involves using a combination of chemotherapy and immunotherapy. This combination has been established as the preferred treatment based on the positive outcomes observed in phase III clinical trials, specifically in terms of improved survival rates, response rates, and the duration of the response [60]. The common approach for the treatment of newly diagnosed stage IV NSCLC is to combine platinum-based chemotherapy with PD-(L)1 inhibition, irrespective of tumor PD-L1 status and in the absence of any contraindication to ICI [49]. Each combination presents its own balance of efficacy and safety, offering valuable options for first-line treatment in this patient population.

### Pembrolizumab + chemotherapy

In the KEYNOTE-189 trial involving newly diagnosed metastatic nonsquamous NSCLC patients without EGFR/ALK mutations, chemoimmunotherapy with pembrolizumab, pemetrexed, and platinum-based chemotherapy resulted in a lower risk of death (HR: 0.60), improved 5-year survival (19.4% vs. 11.3%), and better disease control (HR: 0.50, 5-year PFS: 7.5% vs. 0.6%) compared to the placebo group. Interestingly, these benefits remained consistent across various levels of PD-L1 expression in tumor cells. In conclusion, the 5-year results from the KEYNOTE-189 study strongly support the use of pembrolizumab in conjunction with pemetrexed and platinum-based chemotherapy as the standard therapy in previously untreated nonsquamous mNSCLC patients, providing substantial and long-lasting enhancements in overall and progression-free survival [6, 61]. In the KEYNOTE-407 study on untreated metastatic squamous NSCLC patients, 559 participants were randomly divided into two groups. One group received pembrolizumab with chemotherapy (paclitaxel/nab-paclitaxel + carboplatin), while the other received a placebo with chemotherapy. After 56.9 months, the pembrolizumab group showed significant improvements in both OS and PFS. Notably, the 5-year OS rate was 18.4% with pembrolizumab, nearly double that of the placebo group at 9.7%. The study reported manageable toxicity levels with a 3-year OS rate of 69.5%. Pembrolizumab with chemotherapy is now the standard first-line treatment for untreated metastatic squamous NSCLC, regardless of PD-L1 expression, showcasing significant survival benefits and becoming the preferred first-line option [7, 62].

### Cemiplimab + chemotherapy

Cemiplimab, an anti PD-1 inhibitor, was studied in the EMPOWER-Lung 3 trial in patients with advanced mNSCLC without EGFR, ALK, or ROS1 abnormalities. A total of 466 patients were randomly assigned (2:1 ratio) to receive cemiplimab with specific platinum-doublet chemotherapy or a placebo with chemotherapy. After 28.4 months, the cemiplimab group had a median OS of 21.1 months, compared to 12.9 months in the chemotherapy-only group (HR 0.65), with a median PFS of 8.2 months for cemiplimab vs. 5.5 months for chemotherapy alone (HR 0.55). Overall, the combination of cemiplimab with chemotherapy improved OS, PFS, and overall response rate (ORR) in patients with advanced NSCLC, irrespective of histological subtype and PD-L1 expression levels, but with a higher incidence of TRAEs [63]. In the KEYNOTE-407 study, the median OS in patients with mNSCLC treated with pembrolizumab plus chemotherapy was 17.2 months. These data were derived from a median follow-up period of 40.1 and 56.9 months [64]. In the 2-year analysis of the EMPOWER-Lung 3 studies [63], the HR for overall survival OS in patients with squamous NSCLC was 0.61, while in the final analysis of KEYNOTE-407, the HR for OS in patients with metastatic squamous NSCLC was 0.71 [65]. Cemiplimab stands out as the second PD-(L)1 inhibitor that has demonstrated efficacy in advanced NSCLC, either on its own or in combination with chemotherapy, regardless of whether the cancer is of squamous or nonsquamous histology [12].

### Atezolizumab plus chemotherapy ± bevacizumab

In addition to pembrolizumab and cemiplimab, there are other approved treatment options available. One such alternative is the combination of atezolizumab with carboplatin and nab-paclitaxel chemotherapy for mNSCLC lung cancer. This approach was studied in the IMpower130 trial, which aimed to assess the efficacy and safety of this combination compared to chemotherapy alone as a first-line treatment. The study involved 724 patients. The co-primary goals focused on assessing PFS and OS in the intention-to-treat population that lacked EGFR or ALK mutations. The trial ran from 2015 to February 13, 2017. The results showed significant improvements in both OS (18.6 vs. 13.9 months; HR 0.79) and PFS (7.0 vs. 5.5 months; HR 0.64) with atezolizumab plus chemotherapy compared to chemotherapy alone. The most common grade 3 or higher TRAEs were myelosuppression-related events. These findings suggest that the combination of atezolizumab with carboplatin and nab-paclitaxel chemotherapy may be a valuable first-line treatment option for patients with mNSCLC lung cancer, and it was generally well tolerated [9]. The IMpower150 trial assessed the combination of atezolizumab with bevacizumab and chemotherapy as a first-line treatment for mNSCLC patients, including those with varying levels of PD-L1 expression and previous EGFR or ALK alterations. After 1,202 patients were enrolled, they were divided into three groups: ACP (atezolizumab-carboplatin-paclitaxel), ABCP (atezolizumab-bevacizumab-carboplatin-paclitaxel), and BCP (bevacizumab-carboplatin-paclitaxel). Results revealed a median OS of 19.0 months for ACP vs. 14.7 months for BCP (HR 0.84), with ABCP also showing a longer OS (19.5 months) compared to BCP (HR 0.80). Exploratory analyses suggested a longer OS with ACP and ABCP in PD-L1–high and PD-L1–positive subgroups, while PD-L1–negative subgroups had similar OS. The safety profile remained consistent. While ACP showed a numerical OS improvement over BCP, it was not statistically significant. However, with additional follow-up data, further OS improvement was observed with ABCP. This study supports the combination of immunotherapy, chemotherapy, and angiogenesis inhibitors like bevacizumab as an effective treatment for certain lung cancers, with FDA approval as an alternative option for advanced nonsquamous NSCLC patients without driver mutations [66]. In addition to FDA approval this first-line treatment option is also approved by the EMA [48]. The advantage of ABCP over BCP in terms of PFS was evident even in patients who had liver metastases at the beginning of the study. In the KEYNOTE-189 trial, patients with liver metastases had positive outcomes with pembrolizumab and chemotherapy, suggesting that this combination may also be a viable treatment option for this subset [67, 68].

### Dual ICI

#### Anti-PD1 + anti-CTLA-4

Among the use of a combination chemotherapy and immunotherapy, researchers have investigated the efficacy of using anti-PD-1 and anti-CTLA-4 in different situations related to NSCLC [69]. First, researchers examined anti-PD-1/PD-L1 and anti-CTLA-4 combinations in metastatic melanoma, and found that these combinations generated enduring positive responses, irrespective of PD-L1 expression levels [70]. Researchers in the CheckMate227 trial observed a significant improvement in OS across all levels of PD-L1 expression, including patients with less than 1% expression. This study was a groundbreaking phase III trial evaluating the efficacy of combining nivolumab (anti-PD1) and ipilimumab (anti-CTLA-4) in the treatment of advanced NSCLC. The trial included a total of 1739 patients regardless of their PD-L1 expression. In patients with PD-L1 expression ≥1%, nivolumab plus ipilimumab resulted in a median OS of 17.1 months, a higher objective response rate of 35.9%, and a significantly longer duration of response (median 23.2 months) compared to chemotherapy (OS: 14.9 months, response rate: 30.0%, duration: 6.2 months). In terms of TRAEs, 32.8% of patients experienced grade 3 or 4 events with nivolumab plus ipilimumab, while 36.0% of patients had these events with chemotherapy. Discontinuation due to TRAEs was more common with dual immunotherapy (18%) compared to chemotherapy (9%). The most common immun-related adverse effects observed in individuals receiving nivolumab plus ipilimumab were skin-related issues (occurring in 34% of cases) and endocrine events (experienced by 24% of patients). This study supports the use of dual immunotherapy as a highly effective first-line treatment for advanced NSCLC, offering a substantial improvement in OS and response duration compared to traditional chemotherapy, irrespective of the patient’s PD-L1 expression level [12]. In the CheckMate-227 study, the 5-year overall survival rate for patients with advanced NSCLC with PD-L1 ≥1% was 24% when treated with the nivolumab-ipilimumab combination, as opposed to 14% for those receiving chemotherapy alone. The use of dual ICI demonstrated enhanced OS in both histologic subcategories, with a greater advantage in squamous compared to nonsquamous. Moreover, within the squamous subtype, the benefit was more pronounced in patients with PD-L1 expression less than 1% than for those with PD-L1 expression greater than or equal to 1% in lung cancer [71].

### Dual ICI + Cht

#### Anti-PD-(L)1 + anti-CTLA-4 + chemotherapy

Building on the findings of CheckMate227, researchers in the CheckMate-9LA trial found that adding a short course of two cycles of platinum-doublet chemotherapy to the combination of nivolumab and ipilimumab had a significant impact on overall survival compared to using chemotherapy alone. This combination also showed a favorable risk-benefit profile. Results showed a median OS of 15.6 months with nivolumab/ipilimumab/chemotherapy, compared to 10.9 months in the control group, representing a significant OS improvement (HR 0.66 in favor of the experimental group). Moreover, there were improvements in PFS (6.8 months vs. 5.0 months in the control group, HR 0.70) and a higher overall response rate (38% vs. 25% in the control group) in the experimental group. It is worth noting that the most common grade 3–4 TRAEs included neutropenia (7% vs. 9%) and anemia (6% vs. 14%) [14]. At three-year follow-up, the combination of nivolumab, ipilimumab, and two cycles of chemotherapy maintained significant OS benefit (mOS 15.8 months vs. 11.0 months, HR 0.7) compared to chemotherapy alone in the intent-to-treat population. Additionally, the three-year overall survival rate (3-year OS) was notably higher in the nivolumab-ipilimumab group (27% vs. 19%). In patients with baseline brain metastases, nivolumab-ipilimumab showed impressive efficacy, including a median OS of 19.3 months (HR 0.45), significantly improved systemic PFS (9.7 vs. 4.1 months, HR 0.44), and substantial intracranial PFS benefit (11.4 vs. 4.6 months, HR 0.42). These results indicate the efficacy of combination therapy in patients with pretreated baseline brain metastases [72]. The POSEIDON trial investigated the combination of tremelimumab (anti-CTLA-4) with durvalumab (anti-PD-L1) and chemotherapy (T + D + CT) and durvalumab with chemotherapy (D + CT) versus chemotherapy alone (CT) as the initial treatment for mNSCLC in patients with EGFR/ALK wild-type tumors. Results showed that D + CT significantly improved PFS over CT alone (HR 0.74; median PFS 5.5 vs. 4.8 months), while T + D + CT significantly enhanced both PFS (HR 0.72; median PFS 6.2 vs. 4.8 months) and OS (HR 0.77; median OS 14.0 vs. 11.7 months) compared to CT alone. However, the improvement in OS for D + CT versus CT did not reach statistical significance (HR 0.86; median OS 13.3 vs. 11.7 months). The 24-month OS rates were also significantly higher with T + D + CT (32.9% vs. 22.1%). TRAEs of maximum grade 3-4 were observed in 51.8% for T + D + CT, 44.6% for D + CT, and 44.4% for CT.

In summary, the combination of durvalumab with chemotherapy enhanced PFS compared to chemotherapy alone, and the addition of a short course of tremelimumab to durvalumab and chemotherapy resulted in significant improvements in both OS and PFS compared to chemotherapy, without a significant increase in tolerability issues [15]. This suggests that it may be a promising new option for the first-line treatment of mNSCLC. It is important to mention that regulatory approval has been granted for the combined use of tremelimumab and durvalumab alongside platinum-based chemotherapy in patients with metastatic NSCLC with no EGFR or ALK genetic alterations. In the Checkmate-227 study, when patients had to stop treatment due to TRAEs, the median OS was 41.5 months, and the 5-year OS rate was 39% [72]. Similarly, in the CheckMate-9LA trial, 48% of patients experienced grade 3–4 TRAEs, and 18% of them had to discontinue treatment. The median OS in this group was 27.5 months, with a 4-year OS rate of 41% [73]. Comparable findings were observed in the POSEIDON trial, where 58% of patients experienced grade 3 TRAEs, and 9.4% had to discontinue treatment [15]. Thus, the safety profile should not be an obstacle to the widespread application of dual ICI, whether with or without chemotherapy, in routine clinical practice.

In the IMpower 131 and POSEIDON trials, the combination of atezolizumab and durvalumab with chemotherapy led to median OS times of 14.2 and 14.0 months, respectively, but no survival benefit over chemotherapy was observed in either trial [15, 74]. Cemiplimab in combination with platinum-based doublet chemotherapy (EMPOWER-Lung 3), the combination of durvalumab, tremelimumab, and platinum-based doublet chemotherapy (POSEIDON), and the combination of nivolumab with ipilimumab (CheckMate 227, specifically for PD-L1≥1% tumors) have obtained FDA approval but are awaiting approval by the EMA [49].

Among the previously mentioned studies with predominantly favorable results, there are a few exceptions where the outcomes were not favorable. In MYSTIC, durvalumab plus tremelimumab did not improve OS or PFS in patients with ≥25% PD-L1 expression. Similarly, in the NEPTUNE trial (which included metastatic NSCLC patients with a blood tumor mutational burden of ≥20 mutations per megabase), durvalumab and tremelimumab did not enhance overall survival compared to chemotherapy [59, 75].

### Role of immunotherapy in NSCLC with driver mutation

Mutations such as EGFR, ALK, KRAS, and other genetic changes (including MET, RET, BRAF, and ROS1) have brought about a significant shift in the way this type of NSCLC is treated. In the era of immuno-oncology, there is growing evidence suggesting that prominent oncogenes have different impacts on the immune microenvironment within tumors, which in turn affects the clinical advantages of using ICIs as a treatment approach.

### EGFR

EGFR-activating mutations, which are common in NSCLC, are treatable with targeted therapies and more prevalent in non-smokers, light smokers, young, Asian, and female patients. ICIs have limited efficacy in EGFR-mutant NSCLC based on data from studies like CA209-012 and Keynote 001, where response rates were lower than in wild-type patients. The ATLANTIC and PACIFIC studies also found that EGFR- or ALK-positive patients had worse outcomes with durvalumab treatment compared to wild-type patients. The IMpower 150 trial showed improved OS in EGFR-mutant patients treated with ABCP compared to BCP, but overall, ICIs alone or with chemotherapy have limited efficacy in EGFR-mutant NSCLC [10, 76, 77].

### ALK

Studies like ATLANTIC and IMMUNOTARGET did not find immunotherapy to be effective. These findings were further supported by retrospective analyses conducted in Massachusetts and a multicenter study in France. In the IMpower150 trial, the combination of chemotherapy with atezolizumab and bevacizumab did not show a significant difference in PFS in ALK-positive patients compared to bevacizumab/chemotherapy (8.3 vs. 5.9 months; HR 0.65; not significant), consistent with the results of IMpower130. In ALK-rearranged NSCLC, ICIs alone do not appear to be promising; chemotherapy remains the standard after ALK tyrosine kinase inhibitors (TKIs) lose efficacy [10, 78].

### KRAS

In advanced NSCLC, KRAS mutations stand out as the most common molecular abnormalities. These KRAS mutations exhibit considerable diversity and involve substitutions at codons 12, 13, or 61. The most frequent of these substitutions which is present in 41% of KRAS-mutant NSCLC cases is KRAS p.G12C. Studies have shown that immunotherapy consistently demonstrates clinical activity that is at least equivalent to that seen in patients who have wild-type KRAS. In a meta-analysis, it was observed that KRAS-mutant patients responded more favorably to ICI treatment when compared to receiving docetaxel monotherapy [79–81].

### BRAF

BRAF mutations, present in approximately 2% of NSCLC cases, with p.V600E being the most common type, have shown clinical efficacy with ICIs in two retrospective cohorts of 39 and 38 patients, including those with p.V600E variants. PFS ranged from 3.0 to 4.1 months, and OS was 13.1 months in the second cohort. Smoking-related factors, such as higher mutational burden and PD-L1 expression, may contribute to increased ICI sensitivity in BRAF mutant NSCLC, similar to KRAS mutations [82, 83].

### ROS1

ROS1 rearrangements occur in approximately 2.5% of lung adenocarcinoma patients, with various fusion partners. Tyrosine kinase inhibitors like crizotinib, entrectinib and lorlatinib are approved for treatment, but the efficacy of ICIs remains uncertain. The IMMUNOTARGET registry, which included six patients, reported a low response rate (17%) and a median OS of 18.4 months in ROS1 fusion-positive NSCLC. Negrao et al suggested in their study that PD-L1 may not be an independent predictor of immunotherapy response [81, 84, 85].

### MET

MET, a receptor tyrosine kinase, is crucial in cell processes and is implicated in NSCLC, with MET exon 14 skipping mutations in 3%–5% of cases and amplifications in 1%–5% of NSCLC patients, effectively treated with drugs like crizotinib, capmatinib, savolitinib, and tepotinib. In the IMMUNOTARGET registry it was observed that the efficacy of ICIs was not influenced by either high PD-L1 expression or a high TMB. If targeted therapy for MET is available, it is advisable to consider its use as a first-line treatment [81, 84, 86–88].

### Her2

HER2 mutations and amplifications are found in 2%–4% of lung adenocarcinomas, often in non-smokers, and are effectively treated with drugs like ado-trastuzumab emtansine (T-DM1) and TKIs. Lung tumors with Her2 amplification have high TMB and low PD-L1, which may explain the reduced efficacy of immunotherapy. In the IMMUNOTARGET study, Her2-mutant patients showed lower ORR, median PFS, and OS, suggesting that chemo-immunotherapy should not be the initial treatment option [81, 89].

### TP53

TP53, a well-studied gene, is vital for cell cycle regulation, DNA repair, and apoptosis. TP53 mutations, when co-occurring with KMT2C, may lead to a better ICI response in patients with advanced NSCLC, while mutations in STK11 and KEAP1 do not notably affect ICI response in this genetic context [90, 91].

### RET

RET rearrangements are found in approximately 1%–2% of patients with NSCLC. Patients with RET fusions are generally never smokers with lung adenocarcinoma and early development of intracranial metastases. The FDA and EMA have granted approval for the use of highly selective RET inhibitors, such as selpercatinib and pralcetinib, specifically for NSCLC cases with RET fusion. While there is a lack of prospective data, existing evidence indicates that NSCLC with RET rearrangements characterized as biologically “cold” tumors do not respond well to ICIs. Therefore, it is advisable to prioritize targeted therapies when they are available for these RET-rearranged NSCLC patients [84, 92].

### NTRK

NTRK gene fusions occur in approximately 0.2% of cases without a clear correlation to sex, age, or smoking history. Larotrectinib and entrectinib are approved for the treatment of these tumors. In NSCLC, NTRK gene fusions exhibit higher TMB and PD-L1 expression than EGFR, ALK, and ROS1 alterations, suggesting a combination of chemotherapy and immunotherapy for comprehensive treatment [93, 94].

## Beyond the first line, rechallenge

When patients cannot receive first-line immunotherapy for any reason or show progression after platinum doublet treatment, and they become eligible for ICI therapy, anti-PD-(L)1 monotherapy is the preferred choice. The FDA and EMA subsequently approved the anti-PD-1 antibody nivolumab and the anti PD-L1 antibody atezolizumab irrespective of PD-L1 status and the anti PD-1 antibody pembrolizumab only in patients with PD-L1 expression. Nivolumab was the first approved agent to show efficacy in patients with advanced and metastatic NSCLC in the second-line and beyond setting. These results are based on two trials, CheckMate-017 and -057. CheckMate-017 evaluated nivolumab at a dose of 3 mg/kg Q2W compared versus docetaxel 75 mg/m^2^ Q3W in 272 patients with advanced or metastatic squamous cell lung carcinoma. Both OS (9.2 vs. 6 months; HR: 0.59) and PFS (3.5 vs. 2.8 months; HR: 0.62) showed a significant improvement for the nivolumab arm independent of PD-L1 status [95]. The CheckMate-057 trial also demonstrated an OS benefit (12.2 vs. 9.4 months; HR: 0.73) of nivolumab (3 mg/kg Q2W) over docetaxel (75 mg/m^2^ Q3W) showed inferiority in terms of PFS (2.3 vs. 4.2 months), but it was superior at 1 year (19% vs. 8%; HR: 0.92). The higher the PD-L1 expression the greater the survival benefit [96]. Based on these data the drug received approval from both medical agencies in both histologic subtypes. Another optional anti-PD-1 agent for second-line treatment is pembrolizumab which was found to be superior to docetaxel. In the KeyNote-010 trial the effects of pembrolizumab at 2 or 10 mg/kg Q3W were compared to docetaxel (75 mg/m^2^ Q3W) in patients with advanced or metastatic NSCLC with PD-L1 expression (≥1%). Both doses of pembrolizumab demonstrated an OS benefit (10.4 months for pembrolizumab 2 mg/kg (HR: 0.71), 12.7 months for pembrolizumab 10 mg/kg (HR: 0.61), and 8.5 months for docetaxel) but no PFS benefit. In a subgroup of patients with higher PD-L1 expression (TPS ≥50%), the PFS was also significantly better (HR: 0.59) [97]. Based on the above data pembrolizumab was also approved by the FDA and EMA for second-line use in PD-L1 positive (≥1%) patients. The third agent which is an anti PD-L1 antibody and therefore slightly different from the other two agents was atezolizumab. The OS (13.8 vs. 9.6 months; HR: 0.73) superiority and PFS non-inferiority over docetaxel was demonstrated in the OAK trial [98]. Based on these results, atezolizumab was also approved by the european (EMA) and american (FDA) medical agencies. In selected patients rechallenge with ICI (especially with pembrolizumab) may be an option if the reason for previous discontinuation was not disease progression or toxicity and clinical benefit was achieved during ICI administration [54, 99].

## Discussion

**FIGURE 1:**
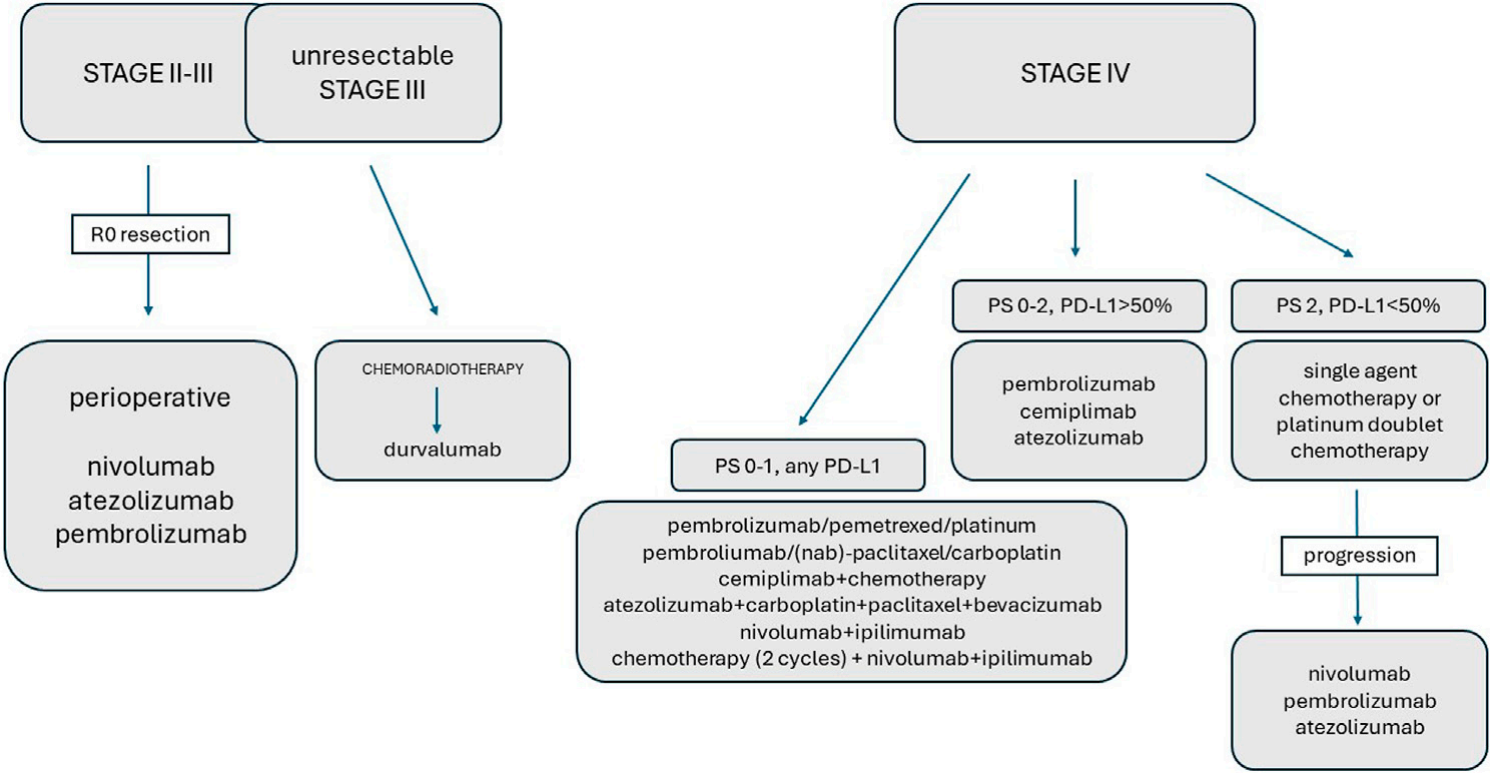
The role of immunotherapy in the treatment of NSCLC.

The future landscape of NSCLC immunotherapy is promising and continually evolving, revolutionizing the treatment paradigm for this aggressive disease. Immunotherapy has emerged as a game-changer, offering new hope and improved outcomes for these patients. ICIs targeting PD-1/PD-L1 have become a cornerstone of this treatment. They have shown significant improvements in OS, PFS, and durable responses compared to conventional chemotherapy. Initially limited to patients with high PD-L1 expression, ICIs are now being considered for a broader patient population, including those with low or no PD-L1 expression. The future lies in combining immunotherapeutic agents with other immunotherapies, chemotherapy, targeted therapies, or even radiation. These combinations aim to enhance the immune response and address tumor heterogeneity and resistance mechanisms. Research into novel biomarkers beyond PD-L1 and TMB is ongoing. Identifying more precise predictors of response to immunotherapy will enable better patient selection and personalized treatment strategies. Research is underway to develop and validate new checkpoint inhibitors targeting different immune checkpoints other than PD-1/PD-L1 and CTLA-4. These could potentially offer improved responses and reduced resistance. The role of immunotherapy is expanding beyond advanced stages. The neoadjuvant approach with an adjuvant ICI combination may be the best option for patients who have no contraindication to immunotherapy, but another ICI + chemotherapy combination may be better than standard-of-care chemotherapy alone even in the adjuvant setting. It is anticipated that ICI will become a mandatory part of the perioperative treatment of resectable NSCLC and ctDNA level measurement may become critical in deciding whether or not patients require adjuvant therapy or not (with or without prior neoadjuvant treatment). This is being explored in the adjuvant setting after surgery or in combination with chemoradiotherapy for locally advanced disease, potentially preventing recurrence. These therapeutic possibilities are summarized in Figure 1. Resistance mechanisms limit the efficacy of immunotherapy. Strategies to overcome this hurdle include combination therapies, the development of novel drugs, and a better understanding of the tumor microenvironment. The landscape is shifting to a more patient-centered approach that emphasizes quality of life, management of treatment-related toxicities, and addressing the unique needs of each patient. Patients with BRAF or KRAS/TP53 mutations benefit most from ICIs, while those with EGFR or ALK/ROS1 rearrangements show lower PD-L1 and mutational burden, leading to ICI resistance. Understanding the genomics of NSCLC will help select ICI candidates. Targeting immunosuppressive mechanisms alongside oncogene signaling may sensitize NSCLC to ICIs. Tumors with driver mutations are diverse, therefore they require precision medicine. Frontline TKIs are preferred to ICIs, with chemo-immunotherapy as an alternative. Combinations of targeted therapy and ICIs are being studied. Broader oncogenic factors should be considered in future NSCLC ICI studies. Ongoing clinical trials are exploring new treatment modalities, innovative drug combinations, and novel therapeutic targets. These trials are crucial for advancing the field and bringing new therapies to the clinic. Despite advancements, the high cost of immunotherapies poses a challenge to accessibility. Efforts to balance innovation and affordability through biosimilars and health policy interventions remain critical. The future of NSCLC immunotherapy is bright, driven by a deep understanding of tumor biology, rapid advancements in technology, and collaborative efforts across the scientific community. As research continues, the goal remains to further improve outcomes, extend survival, and ultimately transform NSCLC into a manageable, chronic condition for more patients.
